# The History of Urinary Stones: In Parallel with Civilization

**DOI:** 10.1155/2013/423964

**Published:** 2013-11-20

**Authors:** Ahmet Tefekli, Fatin Cezayirli

**Affiliations:** ^1^Department of Urology, Bahcesehir University School of Medicine, 34353 Istanbul, Turkey; ^2^Department of Urology, VKF American Hospital, 34365 Istanbul, Turkey

## Abstract

The roots of modern science and history of urinary stone disease go back to the *Ancient Egyptians *and *Mesopotamia*. Hippocrates defined the symptoms of bladder stones. The first recorded details of “*perineal lithotomy*” were those of Cornelius Celsus. Ancient Arabic medicine was based mainly on classical Greco-Roman works. Interestingly, the Fourth Lateran Council in 1215 forbade physicians from performing surgical procedures, as contact with blood or body fluids was viewed as contaminating to men. With Renaissance new procedures could be tried on criminals. The first recorded suprapubic lithotomy was carried out by Pierre Franco in 1561. In 1874, Bigelow developed a lithotrite, which was introduced into the bladder under anaesthesia (called as “*litholopaxy*”). Young was the first to report ureteroscopy (1929). With advances in intracorporeal lithotripsy techniques, ureteroscopy became the treatment of choice for ureteric stones. In 1976, Fernstrom and Johannson established percutaneous access to remove a renal stone. However, with the introduction of the first extracorporeal shock wave machine in 1980, a dramatic change in stone management was observed. Civilization in parallel with scientific developments has brought us to a point where we try not to “cut” our patients for stone disease, as Hippocrates admonishes, but rather manage them with minimal invasive alternatives.

The history of urinary stones almost begins and goes parallel with the history of civilization. The roots of modern science and philosophy go back to the *Ancient Egyptians*, in whom we see the first signs of social and scientific developments. In 1901, the English archeologist E. Smith found a bladder stone from a 4500–5000-year-old mummy in El Amrah, Egypt. Treatments for stones were mentioned in ancient Egyptian medical writings from 1500 BC [1, 2].

The earliest literary quotations to stone disease, describing symptoms and prescribing treatments to dissolve the stone, are observed within the medical texts of *Asutu in Mesopotamia* between 3200 and 1200 BC [1]. And the first descriptions of “cutting for the stone” are found in Hindu and Greek writings. Sushruta (*around* 600 BC) was a surgeon who lived in ancient India and is the author of the book *Sushruta Samhita*, in which he describes over 300 surgical procedures, including *perineal lithotomy* [3, 4]. The formation of bladder stones was also described in these texts as follows. “Bladder stones are normally carried in to the bladder. If the internal channels are not kept clean or unwholesome food is eaten, the mixture of deranged Kapham (phlegm) and urine forms stones. Bigger stones form in the same fashion as the precipitate that occurs after some time when even clear water is kept in a new pitcher.” A vegetarian diet, a urethral syringe of medicated milk, clarified butter, and alkalis were treatment recommendations for stone sufferers in the Ancient India. When these treatments failed, surgery was used, as described in detail in Sushruta's works [4].

Ancient Greeks, who settled down the basis of philosophy and science, did the first remarkable observations and documentations concerning urinary stone disease. Hippocrates (460–377 BC) described diseases of the kidney and defined symptoms of bladder stones. In his famous *Oath of Medical Ethics* for physicians, he underlines “*I will not cut for the stone, but will leave this to be done by practitioners of this work*.” At that time, lithotomy was practiced with only perineal incision by special lithotomists and Hippocrates adamantly stated that wounds of the bladder were lethal [5]. This admonition to physicians about a very risky procedure was to be held for centuries. 

Ammonius of Alexandria (276 BC) was the first person to suggest crushing the stone to facilitate its removal [6]. He stabilized the stone with a hook and then split the stone using a thin blunt-ended instrument. Since he was the first to use the word “*lithotomus*” referring to cutting the stone, he was given that nickname. However, his idea did not gain popularity at that time [6]. 

The first recorded details of “*perineal lithotomy*” were those of Cornelius Celsus (25 BC–40 AD), who lived in Rome and wrote an encyclopedia of medicine (*De Medicina*) [1, 6, 7]. Although he, as a physician, never performed the operation himself, his description of perineal lithotomy was a landmark in the history of urology. This technique, aptly called the “*Operation Minor*” or “*petit appareil*”, was used with very little change, indeed if any, for the next 1500 years. Celsus recommended the procedure to be carried out in spring, between ages of 9 and 14, with the help of two strong as well as intelligent assistants. Calus Plinus Secundus (23–79 AD), Galen (131–200 AD), and Paul of Aegine (625–690 AD) were other outstanding Greek physicians, who were practicing lithotomy, basically as described by Celsus [1, 6, 7].

Ancient Arabic medicine was based mainly on classical Greco-Roman works. Muslim scientists did a great duty in the translation of these classical writings in Latin language and transfering them to the European investigators, who achieved prominent improvements with the Renaissance. Rhazes (841–926 AD) wrote a book on medicine and described perineal lithotomy almost in the same manner as that carried out by Paul of Aegine [1]. 

Shortly afterwards, Albucasis (Ibn Abbas Alzahrawi, 930–1013 AD) from Cordova demonstrated considerable experience in surgery by modifying the technique of lithotomy as practiced by Ancient Greeks [8, 9]. The operation was carried out through a perineal incision down to, then through, the bladder neck to reach the stone and extract it. Comparing the descriptions of the operative technique as carried out during ancient Indian and Greek civilizations, the description given by Albucasis in his book *Al-Tasreef* clearly shows how Albucasis remarkably improved the technique of this operation and reduced its risk [8]. Albucasis also invented a new lithotomy scalpel, called “nechil”, with 2 sharp cutting edges and being a novel instrument not known before him he made a drawing for it. The scalpel, called “Novacu1a” used by the Italian surgeon “Marianus Sanctus” in the 16th century, and the scalpel, used by the English surgeon “Shelsden” in the 18th century, were very close in shape to Albucasis' scalpel [1, 8]. Furthermore, in the ancient and Greco-Roman texts before Albucasis, there is no such emphasis on avoiding the midline perineal incision. That innovation in the technique of perineal cystolithotomy, introduced by Albucasis, was of considerable practical anatomical significance. Albucasis was also the first to use forceps to extract a bladder stone. Before him, extraction of the stone was by an instrument similar to a small spoon that goes around the stone and scoops it out. In Europe, during Renaissance, most of the well-known lithotomists such as the Italian “Marianus Sanctus” (16th century AC), the French “Jack De Beaulieu” (17th century AC), and the English “Shelsden” (18th century AC) were using Albucasis' lateral approach incising on the left side. He is also considered as the first to use a tool to confirm the presence of the stone before proceeding with the perineal cystolithotomy operation. He also introduced the 2-stage bladder stone operation in complicated cases. Albucais' modifications and innovations spread to Europe in the Middle Ages and remained widely adopted until the beginning of the eighteenth century, which witnessed the beginnings of the modern method the suprapubic, instead of the perineal, approach for the removal of bladder stones.

During the medieval period in Europe (1096–1438) there was little activity in the management of stone disease [10, 11]. In this era lithotomists were essentially commercial travelers moving from town to town looking for business and cutting all who came their way [7]. Often uneducated and occasionally dishonest, some were great showmen. The procedure was generally performed in the public without anaesthesia and generally lasted a few minutes [7]. However, lithotomists were held responsible for their bad results and fined accordingly.

In the 14th century, Chauliac (1300–1367), considered as the father of French surgery, wrote the Chirugia Magma, combining surgical influences of the Arabs, the Greeks, and his experiences [12]. He wrote much about stone disease but never performed lithotomy, which was a dangerous operation at that time. Although some separation of surgery from the practice of medicine had begun to develop in early medieval times, this was accentuated in 1215 by the Fourth Lateran Council, a papal edict which forbade physicians (most of whom were clergy) from performing surgical procedures, as contact with blood or body fluids was viewed as contaminating to men [10]. As a result, the practice of surgery was relegated to craft status with training by apprenticeship through guilds. Physicians followed a university-directed program of education, which involved knowledge of the classics and writings of ancient medical authors such as those by Galen, which allowed no independent thought or inquiry. Competition among physicians and surgeons, including the lowest group of surgical practitioners, the barbers, continued until Henry VIII signed a charter in 1540 uniting barbers and surgeons in London. This Guild of Barbers and Surgeons, forerunner of the Royal College of Surgeons, established a regulatory agency for training and certification of surgical practice, which set the stage for legitimizing surgery as a profession [10]. 

With Renaissance (1453–1600), there was a rapid increase in intellectual creativity in many fields. During this period, new procedures could be tried on criminals. As a result, Colot removed stones from a criminal suprapubicly in 1475. Thereafter, the Colot family in France held some kind of a monopoly of lithotomy over 2 centuries [1, 13]. They were members of the College of Surgery and had high reputation. However, the first major scientific improvement since Celsus and Albucasis was done by Farncisco de Romanis in 1520 [1]. He introduced a sound to identify the bladder neck, and the perineal incision was made onto the sound using a broad knife called “*novacula*.” He also used retractors for exploration. His technique was popularized by his student Marius Sanctus, named as “*Marian operation*” or “*Grand Appareil*” [14]. 

Almost at the same period in the 15th century, two Turkish physicians, Sabuncuoğlu Serafettin and Ahi Ahmed Celebi, described independently a new technique of transurethral stone fragmentation and bladder irrigation [15]. They also wrote comprehensive prescriptions to aid stone passage and dissolution in their texts.

Paré (1510–1590), the greatest French surgeon at his time, also wrote a detailed chapter on urinary stone disease and about lithotomy, although he never practiced it [1, 16]. He also wrote long and detailed prescriptions to stone patients in his book. 

The first recorded removal of a calculus by suprapubic lithotomy was also carried out during Renaissance by Pierre Franco in 1561 [13]. Although his patient recovered well, Franco advised others not to follow his example because of the extreme hazards of this approach. 

The first account of an operation performed on the kidney was also around this time during Renaissance. Cardan of Milan opened a lumbar abscess in 1550 and discovered 18 stones [1]. However, there was no further mention of this procedure for many years. 

The next major influence on the practice of lithotomy was Jacques de Beaulieu (1651–1714), who introduced “*lateral lithotomy*” [17]. Thereafter, this method was further perfected and popularized by Ferre Jacques, who performed more than 5000 operations. 

William Cheselden (1722) and John Douglas (1719) were the first to realize that distended bladder mowed upwards and therefore an extraperitoneal approach was possible. However, these two famous friends accused each other of plagiarism, which lasted for many years [1, 7].

Hermann Boerhaave (1668–1738) was one of the most important figures in 18th-century medicine [18]. During the early 18th century the surgical approaches for lithotomy to treat lithiasis had very high risks of complications. In the face of the very common and dangerous complications, the doctors and surgeons actively sought all possible solutions short of surgery and left lithotomy as the last alternative. Boerhaave dedicated a chapter in his “*Institutiones Medicae*” to the treatment of lithiasis of the urinary tract. His recommendations included an increase in liquid intake, a hot bath in order to induce vasodilation, and exercise. Using these methods, Boerhaave felt that stone removal should be achieved, perhaps reflecting both the status of surgery in the early 18th century and an appreciation of the risks of the surgical procedures available. Boerhaave's opinion of lithotomy as a last resort when other approaches failed was “*I think lithotomy is an act of pure faith*” [18].

Although the issue of informed consent has become the concern of medical researchers since the beginning of the 20th century and recently became almost the main issue of medical treatment, we see the medicolegal infrastructure of informed consent concept in the law court archives of the Ottoman Empire during the 16th and 17th centuries [19]. In these informed consents, patients or parents signed that they understood the complications of lithotomy and that they would not complain and bring the case to suit in case of any complication. 

The history of urinary stones is becoming more appealing with the famous persons harboring the disease. Famous historical figures who developed bladder stones include King Leopold I of Belgium, Peter the Great, Louis XIV, George IV, Oliver Cromwell, Benjamin Franklin, the philosopher Bacon, the scientist Newton, the physicians Harvey and Boerhaave, and the anatomist Scarpa [13]. 

Michelangelo, who is thought to have a high-functioning autism, that explains his single-minded work routine, unusual lifestyle, limited interests, poor social and communication skills, and issues of life control, also suffered from urinary stones [20]. Depression and various medical conditions, including gout, renal colic, and urinary stones, did not stop his obsessive working habits. His terminal illness with symptoms of fluid overload suggests that he may have sustained obstructive nephropathy. That this may account for his interest in kidney function is evident in his poetry and drawings. Most impressive in this regard is the mantle of the Creator in his painting of the Separation of Land and Water in the Sistine Ceiling, which is in the shape of a bisected right kidney. His use of the renal outline in a scene representing the separation of solids (Land) from liquid (Water) suggests that Michelangelo was likely familiar with the anatomy and function of the kidney as it was understood at that time [20].

Napoleon Bonaparte and Emperor Napoleon III were suffering from bladder stones and had severe symptoms, probably affecting their decisions and judgments [13]. Today, historians discuss what might have happened in the Russian campaign in 1812 if Napoleon Bonaparte had not had a bladder stone. Similarly, the whole European history might have changed if Napoleon III was treated with modern surgical techniques during the Franco-Prussian War of 1870 [13]. 

By modifying the “primitive lithotrite” developed by Albucasis, Jean Civiale introduced a trilabe, grasping, and fragmenting instrument in 1824 [21]. This can be considered the beginning of the use of lithotripters and “endouralogy” in stone fragmentation. In 1874, Bigelow developed a stronger and harder lithotrite, which was introduced into the bladder with the help of anaesthesia [22]. He filled the bladder, crushed the stones, and evacuated the fragments. This was called “*litholopaxy*.” Suddenly, the mortality rate dropped from 25% to 2.4% [22]. 

Besides the developments in cystoscopic lithotrite, alternative surgical procedures for stone removal were being attempted. Gustav Simon performed the first planned nephrectomy for a fistula in 1869 [23]. In 1873, Ingalls from Boston carried out the first nephrotomy. The first pyelotomy was performed by Heinecke in 1879, and the first nephrolithotomy was carried out in 1881 by Le Dentu [1, 24].Czerny is credited with being the first to suture a nephrotomy incision in 1887 [25]. Kummel and Bardenheuer carried out the first partial nephrectomies for stone disease in 1889 [25]. Max Brodel described the avascular area of the kidney in 1901 [26]. Lower revived interest in pyelolithotomy by suggesting that it may be a safer and easier method for removing renal stones than nephrolithotomy in 1913 [25]. Another important advance in open renal stone surgery was intrasinusally *extended pyelolithotomy*, pioneered by Gil-Vernet in 1965 [27]. Fitzpatrick et al. from England further suggested the combination of extended pyelolithotomy with multiple radial nephrotomies for the treatment of large, complex staghorn stones (1974) [28]. In an experimental study on dogs the authors were able to show that an extended sinus approach to the collecting system of the kidney was associated with no functional or parenchymal loss while the radial paravascular approach was followed by a 20% decrease in function and no significant parenchymal loss; the anatrophic intersegmental nephrotomy caused a 30% functional decrease with a significant parenchymal loss and the bivalve nephrotomy was associated with a 50% loss of function and considerable parenchymal loss and distortion [28].

On the other hand, Smith and Boyce from USA introduced and popularized anatrophic nephrolithotomy for the treatment of staghorn stones in 1967 [29]. This technique has further gained popularity, became treatment of choice for large staghorn stones in experienced hands, and is even applied during laparoscopic approaches [30].

With the increasing use of the Nitze cystoscope and the Hopkins rod-lens system, Young and Mckay (1870–1945) were able to develop the cystoscopic lithotrite. They were also the first to perform (1912) and report ureteroscopy (1929) [31]. Before rigid ureteroscopy, advances in fiber optics led to the development of flexible ureteroscopes. In 1964, Marshall reported his first experience with flexible ureteroscopy using a 3 mm fiberscope [32]. This was followed by Tagaki (1971) and Bush (1970). However, it was not until 1977 that purposeful rigid ureteroscopy was reported independently by Goodman and Lyon et al. [33, 34]. There were significant improvements, proceeding the advances in intracorporeal lithotripsy, in 1980s.

*Electrohydraulic lithotripsy* was the first modern intracorporeal lithotriptor invented in 1954 by Yutkin, an engineer from Kiev [35]. Because he was out of favour with Stalinist government, he was banished and the use of his invention was delayed for at least 10 years, when URAT-1 was displayed and popularized in 1967. Although early users reported severe complications such as perforation, these were followed by successful reports of bladder stone treatment from Europe and USA (1977). The first investigation of *ultrasound* for the destruction of urinary stones was undertaken by Mulvaney in 1953, and Kurth applied it to renal stones in 1977 [36]. The development of *laser* for the fragmentation of ureteral calculi was initiated in 1986 [35]. Significant advances in laser fibers and power generation systems have propelled laser lithotripsy, in many practitioners' hands, as the treatment of choice for ureteral stones. The newest technique approved for the fragmentation of renal, ureteral, and bladder calculi is *pneumatic lithotripsy* [35]. The first pneumatic device, the Lithoclast, was designed by a Swiss company in 1992 [35]. Today, with the advances in flexible ureteroscopes and laser fibers, even stones in the renal calices can be treated by ureteroscopy (retrograde intrarenal surgery). 

Improvements in intracorporeal lithotripsy also allowed renal stones to be treated by percutaneous renal surgery. Rupel and Brown removed a stone in 1941 through a nephrostomy tract that had previously been established surgically [37], and Trattner in 1948 used a cystoscope to examine the renal collecting system at open renal surgery [38]. Goodwin et al. were the first to place a nephrostomy tube to a grossly hydronephrotic kidney to provide drainage in 1955 [39]. It was not until 1976 that Fernstrom and Johannson established percutaneous access with specific intention of removing a renal stone [40]. Advances in endoscopes and other instruments allowed urologists to refine the percutaneous nephrolithotomy technique during 1970s and large series were reported in 1980s [40]. 

However, with the introduction of the first ESWL machine, Dornier HM-3, in 1980, a dramatic change in stone management was observed [41, 42]. Probably, this was the outstanding invention in the management of urinary stones. The US Food and Drug Administration approved the use of ESWL machines in 1984, and thereafter it was used widespread all over the world [43]. However, the limitations of this machine are underlined in recent studies, and ureteroscopy and percutaneous nephrolithotomy gained the position they deserve in current treatment guidelines. 

All these improvements in the management of urinary stone disease have prevented renal damage and related renal failure due to stone to a great extent. Currently, urinary stone disease is not a major risk factor for chronic renal disease in developed countries. 

With the subsequent developments in endourology (ureteroscopy, percutaneous surgery, and ESWL) there is an ongoing search for even less invasive treatments. And civilization in parallel with scientific developments has brought us to a point where we try not to “cut” our patients for stone disease, as Hippocrates admonishes, and rather manage them with minimal invasive alternatives. Currently, open surgery is performed in less than 4% of patients with urinary stones in reference centers [44].
